# Spending on vegetable and fruit consumption could reduce all-cause mortality among older adults

**DOI:** 10.1186/1475-2891-11-113

**Published:** 2012-12-19

**Authors:** Yuan-Ting Lo, Yu-Hung Chang, Mark L Wahlqvist, Han-Bin Huang, Meei-Shyuan Lee

**Affiliations:** 1 Graduate Institute of Life Sciences, National Defense Medical Center, Taipei, Taiwan; 2 Division of Health Policy Translation, Institute of Population Health Sciences, National Health Research Institutes, Taipei, Taiwan; 3 Division of Preventive Medicine and Health Services Research, Institute of Population Health Sciences, National Health Research Institutes, Taipei, Taiwan; 4 School of Public Health, National Defense Medical Center, Taipei, Taiwan; 5 Monash Asia Institute, Monash University, Melbourne, Victoria, Australia; 6 Department of Epidemiology and Preventive Medicine, Monash University, Melbourne, Victoria, Australia

**Keywords:** Food expenditure, Mortality, Elders, Vegetables

## Abstract

**Background:**

Few studies have evaluated the linkage between food cost and mortality among older adults. This study considers the hypothesis that greater food expenditure in general, and particularly on more nutritious plant and animal-derived foods, decreases mortality in older adults.

**Methods:**

This study uses the 1999–2000 Elderly Nutrition and Health Survey in Taiwan and follows the cohort until 2008, collecting 24-hr dietary recall data for 1781 participants (874 men and 907 women) aged 65 y or older. Using monthly mean national food prices and 24-hr recall, this study presents an estimate of daily expenditures for vegetable, fruit, animal-derived, and grain food categories. Participants were linked to the national death registry.

**Results:**

Of the 1781 original participants, 625 died during the 10-y follow-up period. Among the 4 food categories, the fourth and fifth expenditure quintiles for vegetables and for fruits had the highest survival rates. After adjusting for co-variates, higher (Q4) vegetable and higher fruit (Q4) food expenditures referent to Q1 were significantly predictive of reduced mortality (HR = 0.55, 95% CI: 0.39-0.78 and HR = 0.64, 95% CI: 0.42–0.99, respectively) and the risk decreased by 12% and 10% for every NT$15 (US$0.50) increase in their daily expenditures. Animal-derived and grain food spending was not predictive of mortality.

**Conclusion:**

Greater and more achievable vegetable and fruit affordability may improve food security and longevity for older adults.

## Introduction

The gains in public health achieved over the past few decades are currently threatened by the global economic and food crises, with young children, women, and older adults in the developing world likely to be disproportionately affected [1]. Poorer households facing food insecurity may be in fear or running out of food or money to buy food and face food scarcity because of economic decline. In many cases, they purchase cheaper foods than usual or simply go without food for a day or more for economic reasons [2]. Some older people are at risk of malnutrition because of food insufficiency, and have lower mean intakes of various nutrients and lower intakes of vegetables and meat and lower dietary variety [3]. Wealthy households have higher expenditures on animal-source foods, vegetables, and fats and oils compared to moderately and severely food-insecure households [4]. Solutions to these dilemmas require further studies that merge health economics and nutrition in the field of what might be called “nutritional economics” [5].

Most dietary studies have shown that greater vegetable, legumes, and fruit intakes are linked to reduced risk of all-cause mortality in older populations [6-10]. Studies from Bangladesh show that when people spend more money on rice, child and maternal malnutrition increases [11]. This may be because of a corresponding reduction in the intake of other plant foods, such as fruit and vegetables and animal-derived food [11,12]. However, no study has investigated the relationship between expenditures on various food groups and older adult all-cause mortality in a relatively affluent society such as Taiwan. We hypothesize that the purchase of more expensive, albeit nutritious foods, such as vegetables, fruits, and animal-derived foods might predict a lower risk of mortality. Conversely, the purchase of less expensive commodities such as rice might be linked to higher mortality in older adults. We tested this proposition by assessing the food expenditure profiles of a cohort of older Taiwanese adults followed for 10 y and linked to the National Death Registry.

## Methods

### Study participants

The participants in this study were free-living, older adults aged 65 y or older and involved in the nationally representative Elderly Nutrition and Health Survey in Taiwan (NAHSIT) conducted between 1999 and 2000 [13,14]. A total of 1911 (955 men and 956 women) participants completed a 24-hr dietary recall by interview at home. Participants were excluded if they reported extremely high or low total daily energy intakes (<500 or >3500 kcal/d in women, <800 or > 4200 kcal/d in men, n = 126), or if their diets comprised only liquid (mainly milk, n = 2). The remaining participants were linked to the National Death Registry up to December 31, 2008. Two participants were excluded because of incorrect death records, leaving 1781 participants eligible for the final analysis [6,15].

### Daily food expenditure

This study uses 24-hr dietary recall data collected on the survey day to calculate participants’ intakes of specific foods [13]. The data included 3646 foods. For food price assignment, we combined, categorized, and encoded similar food items as 843 foods. Food items were categorized according to the same or similar names, similar nutrient profile, comparable food physical properties, and similar ingredients irrespective of preparation. Each food was priced at the cost per 100 g, as purchased. The cost of each food was determined from the Taiwanese Council of Agriculture, databases from the Agriculture and Food Agency, Poultry and Livestock Products Current Trade, and the Fisheries Agency from January 1999 to December 2000 expressed in retail prices (New Taiwan Dollars, NTD) [16-18]. All foods were matched with the national average monthly retail price (NTD/100 g) based on participants’ interview dates. For foods not included in these sources, prices were obtained from a widely available national supermarket chain with consistent prices across Taiwan. In this case, regular 2009 prices listed were used without discount. The supermarket prices of these goods were then deflated according to the Consumer Price Index for 1999–2000. For the remaining foods without a known cost, proxy prices were used based on the lowest food prices for the same food classification, or the lowest food prices for products with similar ingredients. In total 215 of the 843 foods used this approach. Food costs did not include alcohol, drinking water, or condiments. The food expenditure of participants was generated from these food costs with the assumption that they ate at home. Previous research has presented a detailed description of food price calculation [19]. Food expenditures were assigned to items in 1 of 4 categories: vegetables, consisting of vegetables, beans, soybeans, and soybean products; fruits, consisting of fruit and fresh fruit juices; animal-derived foods, consisting of chicken, duck, pork, beef, goat, fish, and shellfish, eggs, and dairy; and grain foods, consisting of rice and rice products, wheat and flour products, starchy roots and stems, dry beans, and dry bean products. The daily food expenditure for each participant was derived for each of these 4 categories (Table 1).

**Table 1:** Survival status by quintile of daily food category expenditure (NTD)*

		**Deceased (n = 625)**		**Survival (n = 1156)**		***P* ^†^**
**Food category**	**Median**	**n**	**%**	**n**	**%**	
**Vegetables**						
Q1	5.02	165	48.0	191	52.0	0.009
Q2	11.0	126	36.2	230	63.9	
Q3	17.1	122	35.2	234	64.8	
Q4	24.6	101	29.7	255	70.3	
Q5	38.6	111	31.5	246	68.5	
**Fruits**						
Q1	0.00	322	43.3	470	56.7	<0.001
Q2	5.46	97	38.3	150	61.7	
Q3	13.2	68	30.3	179	69.7	
Q4	23.5	66	27.8	181	72.2	
Q5	44.0	72	28.3	176	71.7	
**Animals-derived Foods**						
Q1	7.71	122	37.5	234	62.5	0.264
Q2	20.6	131	38.1	225	61.9	
Q3	33.6	114	29.0	242	71.0	
Q4	50.6	134	36.6	222	63.4	
Q5	80.2	124	38.1	233	61.9	
**Grains**						
Q1	3.77	142	40.5	214	59.5	0.523
Q2	6.26	126	34.4	230	65.6	
Q3	8.63	121	32.0	235	68.1	
Q4	11.4	123	36.3	233	63.7	
Q5	20.5	113	35.6	244	64.4	
**Total food expenditure**						
Q1	33.8	135	40.1	221	60.0	0.255
Q2	56.2	141	41.2	215	58.8	
Q3	72.6	127	33.8	229	66.2	
Q4	94.1	111	32.9	245	67.1	
Q5	132	111	32.8	246	67.2	

* Diet expenditure was calculated as energy-adjusted dietary spending.

^†^Differences across quintile were tested with chi-square test.

### Co-variates

A medical history was obtained through a face-to-face interview and physical examination. Non-dietary variables were assessed using baseline NAHSIT questionnaires. Daily physical activity was measured as the number of metabolic equivalents (METs) calculated from exercise and leisure time activities, based on activity duration, frequency, and intensity and number of flights of stair climbed [6]. Several co-variates were considered, including: gender, age (y), ethnicity (Fukienese, Hakka, mainlander, indigenous), personal education (illiterate, primary and below, secondary education and above), living arrangement (living alone, living with spouse, personal income (<5000, 5000–19,999, ≥20,000 NTD/month), smoking (yes, no), alcohol drinking (yes, no), perceived health status (good, fair, poor), physical activity (<1.5, 1.5-3, >3 MET/day), chewing ability (satisfactory, unsatisfactory), body mass index (<18.5, 18.5-23.9, 24–26.9, ≥27 kg/m^2^), and energy intakes (kcal/day). Type 2 diabetes mellitus (yes, no) was as self-reported. The dietary diversity score (DDS), a simple index for dietary quality, ranged from 0–6 [6].

### Statistical analysis

The 4 categories of daily food expenditures were classified into quintiles of energy-adjusted dietary spending (NTD) using the residual method for energy adjustment [20]. Categorical variables were compared using chi-square tests. Cox proportional-hazards models were used to examine the relationship between food expenditures and mortality. The hazard ratios (HRs) and 95% confidence intervals (CIs) across quintiles for each food category expenditure were compared to those of the lowest quintile. The 3 food category expenditures for vegetables, fruits and grains and total food expenditure were modeled simultaneously with the assumption that total food spending was fixed, and with animal-derived foods excluded given their significantly higher cost. This allowed the interpretability of the estimates of the other 3 categories of food in the model. In other words, with fixed total food expenditure, one category must be substituted for another. In this case, changes in animal-derived foods expenditure would take preeminence, and the exclusion of the category enables evaluating the interplay between vegetables, fruits, and grains, and their effects on mortality. In addition, this study considers food category expenditures as continuous variables to assess the survival consequences of each NT$15 per day, equivalent to US$0.50, increment in various categories, considering other food category expenditures and total food expenditure.

In the multivariable analyses, covariates included: gender, age (y), ethnicity (Fukienese, Hakka, mainlander, indigenous), personal education (illiterate, primary and below, secondary education and above), living arrangement (lived alone, lived with spouse), personal income (<5000, 5000–19,999, ≥20,000 NTD/month), smoking (yes, no), alcohol drinking (yes, no), health status (good, fair, poor), physical activity (<1.5, 1.5-3, >3 MET/day), chewing ability (satisfactory, unsatisfactory), body mass index (<18.5, 18.5-23.9, 24–26.9, ≥27 kg/m^2^), diabetes mellitus (yes, no), and energy (kcal) as well as DDS. Except for DDS, this constituted model 1.

All analyses were conducted using SAS statistical software (version 8.0, 1999, SAS Institute Inc, Cary, NC). SUDAAN (version 9.0, 2004, Triangle Park, NC) was used to account for the sampling design. A value of *p* < 0.05 was considered significant.

### Ethics

The protocol of this study: was approved by the ethics committees of both the Academia Sinica and the National Health Research Institutes in Taiwan. All participants provided signed informed consent.

## Results

A total of 625 (35.9%) participants died during the 10-y follow-up. Table 1 shows the survival status in relation to each of the 4 food category expenditures. The deceased in the highest 2 quintiles of vegetable expenditures were proportionately lower (*p* = 0.009). However, almost 50% of the older adults in the first quintile were deceased. The top 2 quintiles for fruit expenditures have significantly greater survivorship than the lower expenditure quintiles (*p* < 0.001). The differences for animal-derived foods, for grains, and for total food expenditure are not significant.

The cumulative death rates were 68.1 and 39.9 per 1000 person-years in the first and the fifth quintiles of vegetable expenditures, respectively. The lowest rate was 36.3 in the fourth quintile (Table 2). Participants aged 80 y or older, indigenous, or who lived alone were more likely to have lower expenditures on vegetables, and gender, education, and income produced no significant differences. Participants whose chewing ability was satisfactory were more likely to have higher vegetable expenditures; however, perceived health status and physical activity were not significantly associated. The findings for fruits were similar to those for vegetables (data not shown).

**Table 2:** Participant characteristics by quintile of daily vegetable expenditure

		**Quintiles of daily vegetable expenditure (NTD)**										
	**Q1**		**Q2**		**Q3**		**Q4**		**Q5**		***P* ^†^**	
**Characteristics**	**n**	**%**	**n**	**%**	**n**	**%**	**n**	**%**	**n**	**%**		
Cumulative death rate*	**68.1**		**47.6**		**44.8**		**36.3**		**39.9**		
**Gender**											
Male	157	17.6	168	18.1	168	13.9	180	23.0	201	22.1	0.214
Female	199	20.2	188	20.2	188	21.4	176	19.6	156	18.6	
**Age, y**											
65-69	122	14.0	142	19.6	153	21.5	145	22.3	145	22.5	0.003
70-74	107	17.5	129	22.6	109	19.8	119	19.4	126	20.7	
75-79	80	20.3	58	15.8	57	16.6	56	2.4	61	21.0	
≥80	47	26.2	27	15.3	37	22.9	36	21.5	35	14.1	
**Ethnicity**											
Fukienese	213	18.8	239	20.4	221	20.1	223	20.9	211	19.9	<0.001
Hakka	31	19.2	27	13.3	52	24.3	41	22.9	39	20.2	
Mainlander	54	17.4	55	18.9	62	19.4	61	22.0	75	22.4	
Indigenous	58	35.3	34	17.8	21	10.7	30	16.2	32	20.0	
**Education**											
Illiterate	130	20.2	122	19.4	122	20.5	131	20.4	112	19.7	0.955
Primary and below	155	17.7	164	19.0	163	20.6	155	23.3	154	19.5	
Secondary and above	69	18.4	70	19.4	69	19.0	69	19.7	90	23.4	
**Living arrangement**											
Lived alone	145	23.9	114	17.4	111	20.5	116	20.0	104	18.3	0.029
Live with spouse	211	16.5	240	19.9	244	20.3	238	21.9	251	21.5	
**Personal income (NTD/month)**											
<5000	238	20.0	231	20.9	200	20.3	184	18.2	207	20.7	0.247
5000-19,999	70	17.3	85	18.0	98	20.4	103	25.2	84	19.2	
≥20,000	22	13.8	21	15.2	38	23.2	36	28.7	33	19.1	
**Chewing ability**											
Unsatisfactory	147	20.1	128	19.9	129	22.7	122	17.9	118	19.5	0.033
Satisfactory	195	17.1	222	18.9	222	19.4	226	23.4	234	21.2	
**Perceived health status**											
Good	118	16.2	122	17.5	145	21.4	143	22.5	156	22.3	0.144
Fair	157	19.2	165	19.4	161	19.6	167	22.1	153	19.6	
Poor	70	23.0	63	22.3	47	20.1	40	16.1	46	18.5	
**Diabetes mellitus**											
No	303	18.6	312	19.0	308	20.4	307	21.2	316	20.8	0.880
Yes	47	20.6	41	19.8	44	19.8	44	22.0	37	17.8	
**Physical activity (MET/day)**											
<1.5	237	20.7	225	19.8	209	20.1	206	20.7	202	18.7	0.193
1.5-3	42	20.0	37	16.6	38	17.5	43	25.6	40	18.4	
>3	76	14.8	93	18.7	107	21.5	105	20.8	115	24.1	
**Smoking**											
No	280	18.7	278	18.4	286	20.7	283	21.8	277	20.5	0.803
Yes	76	19.6	78	22.0	70	19.0	73	19.5	80	20.0	
**Alcohol drinking**											
No	297	19.9	285	19.2	280	20.0	281	20.9	285	20.1	0.207
Yes	57	14.0	71	19.1	76	22.2	74	23.5	71	21.2	
**Body mass index (kg/m**^**2**^**) ‡**	23.3	0.30	24.1	0.27	23.6	0.26	23.5	0.27	23.6	0.25	
<18.5	27	26.4	12	8.45	15	19.4	19	23.5	19	22.2	<0.001
18.5-23.9	121	19.7	125	17.0	121	21.3	125	20.6	126	21.4	
24-26.9	79	16.4	69	17.7	85	19.1	78	22.8	85	24.0	
≥27	37	14.1	49	21.3	52	22.9	46	20.0	59	21.7	

* Per 1000 person-years.

^**†**^ Differences across quintiles were tested by chi-square for categorical variables.

^‡^ Values are mean and SE.

In the crude model, the HRs for all-cause mortality of the participants significantly decreased as the expenditures of vegetables and fruits increased; however, no such relationship for grains and animal-derived foods was observed. This pattern remains after simultaneous adjustment for total food, vegetables, fruits, and grains expenditures, age, and gender (Table 3). With further adjustments for potential co-variates, the Q4 and Q5 for vegetable expenditure had HR values significantly lower than Q1 for all-cause mortality (Q4, HR = 0.55, 95% CI: 0.39-0.78; Q5, HR = 0.74, 95% CI: 0.50-1.08), and the dose–response relationship was significant. For fruits, Q3 and Q4 expenditures were associated with significantly lower mortality rates compared to Q1 (Q3, HR = 0.62, 95% CI: 0.42-0.92; Q4, HR = 0.64, 95% CI: 0.42-0.99), and the dose–response relationship remained significant in the model 1. With further adjustment for DDS, the Q4 for vegetable expenditure remained significant whereas it did not for fruit. For grains and animal-derived foods, neither the HRs nor the linear trends were significant in the adjusted models.

**Table 3:** Hazard ratio (95% confidence interval) of daily food group expenditures and all-cause mortality (n=1781)

**Food category**	**Quintile of daily food expenditure (NTD)**									***P *for trend**	**Per NTD 15**		**Per NTD 15**^†^	
	**Q1**	**Q2**		**Q3**		**Q4**		**Q5**						
**Vegetable, range**	**<8.20**	**8.21-13.9**		**14.0-20.6**		**20.7-29.7**		**>29.7**						
Crude	Ref.	0.67	(0.51, 0.90)	0.64	(0.46, 0.90)	0.51	(0.38, 0.69)	0.56	(0.39, 0.79)	<0.001	0.83	(0.73, 0.94)	0.80	(0.69, 0.93)
Model 1	Ref.	0.82	(0.61, 1.09)	0.78	(0.52, 1.17)	0.55	(0.39, 0.78)	0.74	(0.50, 1.08)	0.022	0.88	(0.77, 1.01)	0.84	(0.72, 0.99)
Model 2	Ref.	0.87	(0.65, 1.17)	0.83	(0.55, 1.25)	0.58	(0.41, 0.83)	0.79	(0.54, 1.15)	0.041	0.91	(0.80, 1.04)	0.87	(0.74, 1.01)
**Fruit, range**	**0**	**0.10-9.37**		**9.38-17.1**		**17.2-29.5**		**>29.6**						
Crude	Ref.	0.86	(0.65, 1.14)	0.63	(0.46, 0.86)	0.57	(0.43, 0.77)	0.60	(0.43, 0.82)	<0.001	0.86	(0.79, 0.93)	0.84	(0.76, 0.93)
Model 1	Ref.	0.92	(0.73, 1.17)	0.62	(0.42, 0.92)	0.64	(0.42, 0.99)	0.69	(0.45, 1.05)	0.020	0.90	(0.81, 1.22)	0.88	(0.77, 1.00)
Model 2	Ref.	1.02	(0.81, 1.28)	0.76	(0.53, 1.09)	0.78	(0.49, 1.27)	0.80	(0.53, 1.22)	0.219	0.95	(0.87, 1.04)	0.92	(0.82, 1.04)
**Grain, range**	**<5.11**	**5.11-7.40**		**7.50-10.0**		**10.1-14.4**		**>14.4**						
Crude	Ref.	0.81	(0.55, 1.18)	0.72	(0.51, 1.03)	0.86	(0.62, 1.12)	0.84	(0.56, 1.26)	0.485	0.97	(0.79, 1.18)	1.00	(0.82, 1.22)
Model 1	Ref.	0.76	(0.51, 1.12)	0.80	(0.54, 1.20)	0.83	(0.59, 1.18)	0.82	(0.52, 1.30)	0.275	1.00	(0.81, 1.22)	1.02	(0.83, 1.24)
Model 2	Ref.	0.78	(0.53, 1.15)	0.82	(0.56, 1.22)	0.87	(0.62, 1.22)	0.84	(0.54, 1.30)	0.317	1.00	(0.81, 1.22)	1.01	(0.82, 1.24)
**Animal-derived, range**	**<14.6**	**14.7-27.3**		**27.4-41.0**		**41.1-61.6**		**>61.7**						
Crude	Ref.	1.02	(0.69, 1.52)	0.73	(0.49, 1.08)	0.97	(0.60, 1.42)	1.01	(0.69, 1.48)	0.956	1.03	(0.99, 1.07)	1.03	(0.99, 1.08)
Model 1*	Ref.	0.94	(0.67, 1.33)	0.69	(0.44, 1.07)	0.73	(0.50, 1.06)	0.74	(0.44, 1.25)	0.043	0.95	(0.82, 1.09)	0.94	(0.81, 1.10)
Model 2*	Ref.	1.02	(0.72, 1.44)	0.76	(0.47, 1.23)	0.82	(0.55, 1.24)	0.85	(0.48, 1.51)	0.246	0.97	(0.84, 1.12)	0.97	(0.83, 1.13)

Model 1: Adjusted simultaneously for total food, vegetable, fruit, and grain (but not animal-derived foods) expenditures, gender, age (y), ethnicity (Fukienese, Hakka, mainlander, indigenous), personal education (illiterate, primary and below, secondary education and above), living arrangement (lived alone, lived with spouse), personal income (<5000, 5000–19,999, ≥20,000 NTD/month), smoking (yes, no), alcohol drinking (yes, no), health status (good, fair, poor), physical activity (<1.5, 1.5-3, >3 MET/day), chewing ability (satisfactory, unsatisfactory), body mass index (<18.5, 18.5-23.9, 24–26.9, ≥27 kg/m^2^), diabetes mellitus (yes, no), and energy (kcal).

Model 2: Further adjusted for dietary diversity score.

^*^These models for animal-derived foods have also been adjusted for the same variables as for Models 1 and 2, except for grains.

^†^ All subjects who died in the first year of follow-up were excluded.

When treating food expenditure as a continuous variable, every NT$15 increase in daily vegetable or fruit expenditure led to 12% or 10% reductions, respectively, in all-cause mortality, when holding total food and grains expenditures and potential co-variates constant (Table 3). After excluding participants who died in the first year of follow-up, every NT$15 increase in daily vegetable expenditure caused a 16% reduction in all-cause mortality (HR = 0.84, 95% CI: 0.72, 0.99). For fruit, every NT$15 increase in daily fruit expenditure was associated with a 12% reduction in all-cause mortality (HR = 0.88, 95% CI: 0.77, 1.00).

The daily dietary nutrient intakes for those whose vegetable expenditure in Q4 or Q5 were lower in saturated fat and higher in polyunsaturated fat, dietary fiber, Ca, Mg, K and vitamins A, E, B-6, and C (Table 4).

**Table 4:** Daily dietary nutrient intakes by vegetable expenditure^*^

	**Total population**	**Quintiles (range) of daily vegetable expenditure (NTD/day)**					***P*†**
		**Q1 (<8.20)**	**Q2 (8.21-13.9)**	**Q3 (14.0-20.6)**	**Q4 (20.7-29.7)**	**Q5 (>29.7)**	
Energy (kcal)	1626	1574	1667	1586	1677	1621	0.554
Protein (g)	65.2	58.3	62.5	66.0	68.2	70.3	0.001
Total fat (g)	44.1	46.3	44.1	43.4	43.9	43.0	0.737
SFA (g)	13.3	16.0	13.4	13.0	12.6	11.5	0.001
PUFA (g)	13.5	12.0	13.3	13.2	14.1	14.8	0.011
Cholesterol (mg)	207	209	214	222	206	184	0.080
Carbohydrate (g)	215	205	213	218	219	220	0.078
Dietary fiber (g)	18.8	13.7	14.8	16.8	20.9	27.3	<0.001
Fe (mg)	11.5	8.38	9.41	10.5	13.2	15.5	<0.001
Ca (mg)	624	544	556	576	633	798	<0.001
P (mg)	944	862	899	945	965	1039	0.007
Mg (mg)	225	179	196	215	235	294	<0.001
Na (mg)	4520	3499	3753	4245	5118	5831	<0.001
K (mg)	2270	1698	1899	2178	2416	3087	<0.001
Vitamin A (I.U.)	9354	5234	6605	8345	11353	14664	<0.001
Vitamin D (mg)	5.30	4.95	5.57	5.96	5.06	4.67	0.300
Vitamin E (mg)	7.46	5.89	6.46	7.19	8.30	9.23	<0.001
Vitamin B-1 (mg)	1.07	0.96	1.04	1.01	1.13	1.19	0.167
Vitamin B-2 (mg)	1.35	1.43	1.35	1.25	1.29	1.41	0.114
Niacin (mg)	13.4	12.0	12.7	13.4	14.4	14.5	0.050
Vitamin B-6 (mg)	1.11	1.02	1.01	1.05	1.20	1.26	0.006
Vitamin C (mg)	148	105	122	133	171	202	<0.001

^*^All values are presented by mean.

^**†**^ Differences across quintiles were tested by ANOVA.

## Discussion

This study shows that vegetable and fruit expenditures are associated with less risk of death in the elderly in Taiwan. Both of these expenditures are higher than that for grains, but less than that for animal-derived foods. Contrary to the original hypothesis, there was no identifiable association between animal or grain food costs and all-cause mortality.

Vegetables and fruits are important sources of many bioactive components, including polyphenols, carotenoids, folate, and vitamin C [21]. Whether these phytochemicals or micronutrients can explain the association between vegetable and fruit expenditures and all-cause mortality in older adults remains unclear. Research by the International Union of Nutritional Sciences Food Habits in Later life (IUNS-FHILL) study shows that integrated indices of the diet, akin to a Mediterranean diet score, can account for more of the link between diet and mortality than any other food item, except for legumes, or specific nutrients [9 ,10]. Lee et al. found support for this finding for food indices and mortality in Taiwan [6]. A study by the European Prospective Investigation into Cancer and Nutrition (EPIC) showed a small inverse association between the intake of total fruits and vegetables and cancer risk [22] ; a higher intake of fruits and vegetables is associated with a reduced risk of ischemic heart disease mortality in the same cohort [23].

Evidence from rural Indonesia shows that households that spend a greater proportion on plant foods have lower under-5 child mortality [24]. However, no study has focused on food expenditures in relation to older adult mortality. Our study shows that vegetable and fruit expenditures had a protective effect on all-cause mortality in elders. After controlling for potential confounders, the Q4 of vegetables had a 45% lower all-cause mortality compared to those of the lowest quintile, and for fruit had a 36% lower all cause mortality. With adjustment for dietary quality (DDS), the significance of the HR for vegetables was unchanged, and that for fruits diminished. This indicates a greater dependence on dietary diversity or an integrated food pattern for fruit than for vegetable expenditure where the latter appears to have associations with mortality of its own. In some respects, a vegetable mortality link in Taiwan could have been relatively obscure. This is because the general population eats vegetables, and especially green leafy vegetables and soybeans (as myriad forms of tofu) extensively and regularly [6]. Thus, this might not have been a discriminating factor for survival difference. However, seasonality and natural disasters, especially typhoons, can alter vegetable supply dramatically and drive up prices quickly. This in turn makes vegetables unaffordable for the economically marginalized, including some older adults. In the event, vegetable costs in Taiwan are a survival discriminator for older adults. The finding of survival advantage with greater vegetable expenditure makes the prospects of food security for those with limited means and marginal vegetable supplies more achievable.

A possible reason the top quintile of vegetables has a higher HR than the fourth quintile may be the diminishing marginal health benefit of vegetable intakes with their greater consumption, “crowding out” other food commodities. Thus, the Q4 for vegetables may represent optimal food expenditure among Taiwanese older adults for the greatest health benefit (as judged by mortality). The mean for this Q4 for vegetables is 463 g/d for fruit is 377 g/d, which meets the World Health Organization’s recommendations for vegetable and fruit consumption [22]. However, once sufficiency is reached, those who consume more vegetables or fruit (or even other food commodities) might improve their health based on criteria other than mortality, such as disease and wellbeing.

This study cannot rule out the possibility that participants who acquired and consumed more than the food intake analysis identified incurred greater food expenditures. There is the additional question as to whether living longer through eating a more diverse diet costs more. Published findings demonstrate that, for the most part, a food-diverse diet costs more than a less diverse diet [19]. In this case, it costs more to live longer if longevity is dependent on “buying food diversity.” This then begs the question of affordability as reported by Golan et al. [25]. The effect of greater expenditure on vegetables and fruits to reduce mortality may be mediated by increasing dietary diversity. Thus, both food expenditures and dietary quality should be considered in calculating mortality risk. From food and economic policy perspectives, providing people with nutritious and affordable food requires further consideration.

In this study, if older adults increased food expenditure by NT$15 (or US$0.50) per day, with vegetables they would have 12% lower all-cause mortality, and with fruit, a 10% lower all-cause mortality. These values are even higher after the exclusion of those who died in the first year of follow-up. A NT$15 increase for each of these food expenditures should not to be an economic barrier for most older Taiwanese, but may well be for the socioeconomically marginalized. The lack of this increased spending on vegetables and fruits may represent a reluctance, rather than an inability, to expend these amounts. This could be because of food preferences or chewing difficulty, the latter of which appears to be the case in this cohort [15]. This study also adjusts for BMI and diabetes, which reflect energy and metabolic disorders likely to change eating habits, whether they contribute to or are caused by these conditions. However, these situations do not alter the place of vegetable and fruit expenditures in mortality risk.

Households in developing countries, which spend a greater proportion of food expenditure on grain food, tend to have more underweight children and higher under-5-year old child mortality [12,24]. Although this study of older adults in Taiwan shows no adverse effects of grain expenditure on mortality, there was no advantage either. This may be a reflection of the dominant use of polished rice, rather than whole grains, in an age-group where whole grains are likely to confer a health advantage [26]. Other factors also make the expenditure of grain in an older Asian’s diet a special matter. As a common staple food, grain (and especially rice) has cultural significance with its own food, health, and economic system relevance. In Taiwan, the self-sufficiency ratio for rice is almost 100%, and its price is relatively stable [27]. The need for satiation is not usually the problem. However, an older adult’s choice of vegetables or fruits is likely to remain a health consideration across a wide spectrum of economic development and personal circumstances.

This study has focused on food group expenditure rather than food intake in relation to mortality in elders. This can provide information and insight into whether food expenditure and affordability are associated with survival. In addition, it draws attention to the most vulnerable group.

There are some limitations to this study. First, food costs are based on a 1-d food consumption, which might have changed over time. Since individual diets vary from day to day, one 24-hr dietary recall might result in a less precise measurement of usual food intake. However, for any participant, the survey day was random which might lead to non-differential misclassification and might bias overall HR estimates toward the null value [28-30]. Even so, we still see a significant financial effect in relation to mortality. Therefore, with a more precise dietary method, the effect should be more evident. Moreover, Taiwanese older adults are generally relatively stable in their dietary pattern so that the dietary method used may not be a serious limitation [15].

The food prices in this study were derived from national average monthly prices and did not consider regional price variations [31]. In some cases, there are retail food price differences among Taiwanese regions. With the exception of supermarket chain prices, food prices were based on raw food ingredients for domestic use, and did not include the cost margins for preparing food or eating out (which is common in Taiwan). In addition, the food sources may have been home grown or received from friends or relatives. These could result in overestimates or underestimates of actual food costs. Because Taiwan is a relatively affluent country, these findings may not be extrapolated to countries at different stages of economic development or with different types of cultural food. Although the Taiwanese population is predominantly Chinese, there are also significant indigenous populations.

Although some of the barriers to purchasing nutritious food may be overcome, as previously shown in Taiwan [19], vegetables and fruits, which may confer a survival advantage, remain the more costly food categories. This presents a food equity and policy dilemma. This problem may be partly addressed by understanding how household expenditures on various foods affect mortality rates.

Socio-demographic profiles of the population studied in relation to quintiles of vegetable and fruit expenditure are associated with wide divergence for ethnicity (e.g., indigenous Taiwanese) and living arrangements, although not for education or income. The ethnic divergence is correlated with locality and region [6], with Indigenes being more mountainous and east coastal. Since plant food sources may be more local or home-garden and less retail for Indigenes, our generalizations may be correspondingly limited [32]. At the same time, remote communities are less likely to participate in the mainstream food supply as studied by us. There are health risk and outcome variations across Taiwan which may also limit our deductions [33].

In conclusion, vegetable and fruit expenditures are associated with lower mortality among older Taiwanese adults, and may improve survival by contributing to dietary diversity. However, there are limits to the need for these food expenditure measures beyond the higher quintiles of intake. In the Taiwanese economy, the cost of the main grain staple and expenditure on animal produce are not discriminant for mortality. Although the incremental costs are currently modest, an emphasis on national nutrition policy in Taiwan advocating affordable fruits and vegetables should improve food security and longevity among older adults.

## Competing interests

The authors declare they have no competing interests.

## Authors’ contributions

All authors were involved in the study design and collectively wrote the paper; YTL analyzed the data; MLW and MSL coordinated the research; MSL had primary responsibility for the final content. All authors read and approved the final manuscript.
